# Lower Extremity Lymphedema in Gynecologic Cancer Patients: Propensity Score Matching Analysis of External Beam Radiation versus Brachytherapy

**DOI:** 10.3390/cancers11101471

**Published:** 2019-09-30

**Authors:** Won Ick Chang, Hyun-Cheol Kang, Hong-Gyun Wu, Hak Jae Kim, Seung Hyuck Jeon, Maria Lee, Hee Seung Kim, Hyun Hoon Chung, Jae Weon Kim, Noh Hyun Park, Yong Sang Song, Kwan-Sik Seo

**Affiliations:** 1 Department of Radiation Oncology, Seoul National University College of Medicine, Seoul 03080, Korea; wijang92@naver.com (W.I.C.); wuhg@snu.ac.kr (H.-G.W.); khjae@snu.ac.kr (H.J.K.); hyck9004@naver.com (S.H.J.); 2 Cancer Research Institute, Seoul National University, Seoul 03080, Korea; 3 Institute of Radiation Medicine, Medical Research Center, Seoul National University, Seoul 03080, Korea; 4 Department of Obstetrics and Gynecology, Seoul National University College of Medicine, Seoul 03080, Korea; marialee@snu.ac.kr (M.L.); bboddi0311@snu.ac.kr (H.S.K.); chhkmj1@snu.ac.kr (H.H.C.); kjwksh@snu.ac.kr (J.W.K.); pnhkhr@snu.ac.kr (N.H.P.); yssong@snu.ac.kr (Y.S.S.); 5 Department of Rehabilitation Medicine, Seoul National University College of Medicine, Seoul 03080, Korea; rmseo@snu.ac.kr

**Keywords:** lower extremity lymphedema (LEL), gynecologic cancer, external beam radiation therapy (EBRT), risk factor analysis

## Abstract

The goal of this study is to compare the risk of lower extremity lymphedema (LEL) between pelvic external beam radiation therapy (EBRT) and vaginal brachytherapy, and to identify risk factors for LEL in gynecologic cancer patients treated with adjuvant radiation therapy (RT) after radical surgery. A total of 263 stage I–III gynecologic cancer patients who underwent adjuvant RT were retrospectively reviewed. One-to-one case-matched analysis was conducted with propensity scores generated from patient, tumor, and treatment characteristics. Using the risk factors found in this study, high- and low-risk groups were identified. With a median follow-up of 36.0 months, 35 of 263 (13.3%) patients developed LEL. In multivariate analysis, laparoscopic surgery (HR 2.548; *p* = 0.024), harvesting more than 30 pelvic lymph nodes (HR 2.246; *p* = 0.028), and para-aortic lymph node dissection (PALND, HR 2.305; *p* = 0.014) were identified as independent risk factors for LEL. After propensity score matching, the LEL incidence of the brachytherapy group was significantly lower than the EBRT group (*p* = 0.025). In conclusion, high-risk patients with risk factors such as laparoscopic surgery, harvesting more than 30 pelvic lymph nodes, PALND, and adjuvant pelvic EBRT require closer observation for LEL.

## 1. Introduction

Lower extremity lymphedema (LEL) is a complication impairing quality of life in gynecologic cancer patients following radical surgical treatment [1,2]. This complication is quite common, with a reported incidence ranging from 3.6% up to 47.0% [3,4]. If left untreated, lymphedema deteriorates each year [5] and becomes a chronic condition requiring lifelong care with the potential of serious side effects such as malignancy [6,7]. In order to detect and treat lymphedema early, risk factor identification and risk prediction is important. Many studies have been reported to identify risk factors for LEL in gynecologic cancer patients. Adjuvant radiation therapy (RT) is well known to be associated with higher incidence of LEL, regardless of the cancer origin [4,8,9,10], with an approximately 3-fold increased risk in several studies [4,9,10,11,12,13].

However, little is known about the different risks for LEL influence by the two RT modalities, external beam radiation therapy (EBRT) and brachytherapy. Brachytherapy is typically thought to cause less LEL since it targets just the vaginal wall and paravaginal lymphatics. However, few studies have compared the different effects of pelvic EBRT and vaginal brachytherapy on LEL to date [9,14]. Recently, a quality-of-life study by Karabuga et al. [15] demonstrated that brachytherapy was related to a lower risk of LEL compared with EBRT, using a retrospective lymphedema score analysis based on subjective questionnaires. 

In this study, we used an objective and reproducible method to compare the risk of LEL in patients treated with adjuvant pelvic EBRT and brachytherapy. In addition, we analyzed risk factors for LEL in patients treated with adjuvant RT and attempted to identify a high-risk group using the established risk factors.

## 2. Results

### 2.1. Patient Characteristics

Table 1 shows the patient characteristics of the two groups according to RT modality. The EBRT group and brachytherapy group included 221 (84.0%) and 42 (16.0%) patients, respectively. Most of the patients had cervical cancer (47.9%) or endometrial cancer (47.5%). About two thirds of the patients underwent surgery with a laparoscopic approach (64.6%). During surgery, more than 30 pelvic lymph nodes were harvested in 20.5% of the patients, and para-aortic lymph node dissection (PALND) was conducted in 32.3%. More than half (58.2%) of the patients received adjuvant chemotherapy. Compared with the EBRT group, the brachytherapy group included more patients with age ≥ 55 (*p* = 0.007), hypertension (*p* = 0.010), endometrial cancer (*p* < 0.001), early stage (*p* < 0.001), pathologic N0 (*p* < 0.001), laparoscopic surgery (*p* = 0.001), and no adjuvant chemotherapy (*p* < 0.001). 

### 2.2. Overall Incidence of LEL

**Figure 1:**
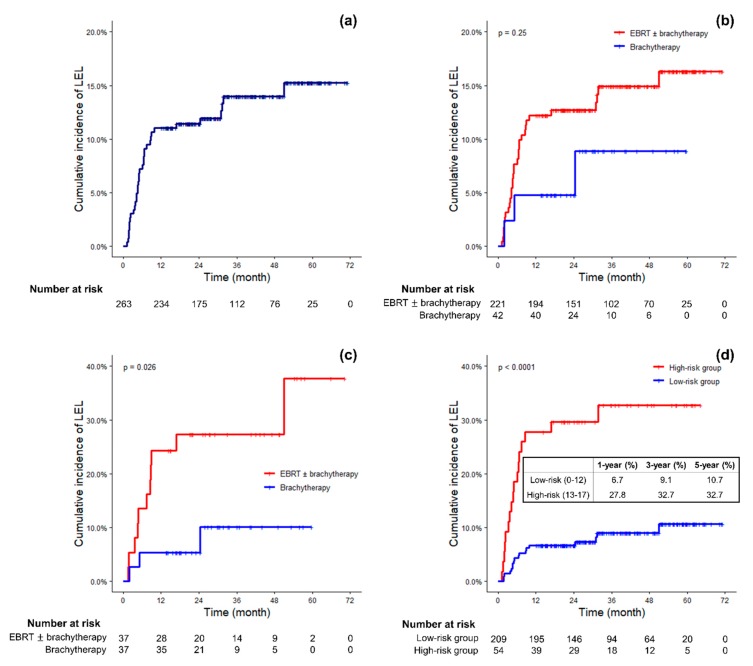
(**a**) Cumulative incidence of lower extremity lymphedema (LEL) in general. (**b**,**c**) Cumulative incidence of LEL according to radiation therapy modality in the entire cohort and in the matched cohort. (**d**) Cumulative incidence of LEL according to risk groups.

During a median follow-up of 36.0 months (12.0–73.0 months), 63 (24.0%) patients reported or were found by gynecologic oncologists or radiation oncologists to have lower extremity edema during or after the course of RT and 49 (18.6%) of them were examined by lymphedema specialists of our institution. Among the 14 patients who were not examined by lymphedema specialists, five patients refused evaluation for lymphedema, two patients were referred to other institutions, two patients were not referred for evaluation of lymphedema because they presented with systemic edema, two patients had only mild symptoms, and three patients were not referred for unknown reasons. Finally, 35 (13.3%) patients met the diagnostic criteria of LEL after circumferential measurements of bilateral extremities. The 1-, 3-, and 5-year cumulative incidence of LEL was 11.0%, 14.0%, and 15.3%, respectively (Figure 1a). Most of the patients (82.9%) were diagnosed with LEL within the first year after surgery. Only one patient was diagnosed after more than 3 years after surgery, for whom it took 51 months to develop LEL postoperatively.

### 2.3. Risk Factors Associated with LEL

In the univariate analysis, harvesting more than 30 pelvic lymph nodes (hazard ratio (HR) 2.106; *p* = 0.037), and PALND (HR 2.391; *p* = 0.010) were significantly associated with a higher incidence of LEL (Table 2). Pelvic EBRT (HR, 1.969; *p* = 0.262) increased the risk of LEL, although it was not statistically significant (Figure 1b). In the multivariate analysis, laparoscopic surgery (HR, 2.548; *p* = 0.024), harvesting more than 30 pelvic lymph nodes (HR, 2.246; *p* = 0.028), and PALND (HR, 2.305; *p* = 0.014) were independently associated with a higher incidence of LEL.

Using propensity scores, 37 pairs of patients from the brachytherapy group and the EBRT group were matched one-to-one, and the matched groups were well balanced (Table 1). After matching, the brachytherapy group showed a significantly lower 3-year cumulative incidence of LEL than the EBRT group (10.1% vs. 27.4%; *p* = 0.026; Figure 1c).

### 2.4. Risk Scores and Risk Groups of LEL Based on the Risk Factors

To predict the risk of LEL, we established an “LEL risk score” using the risk factors that were statistically significant in the multivariate analysis, along with pelvic EBRT which was a significant risk factor after propensity score matching analysis. With the β-coefficients of the identified risk factors, risk scores were derived by multiplying the coefficients with 5 and then rounding to the nearest integer. Thus, a simple scoring algorithm (Table 3) was constructed.

The median risk score was 9, and the cumulative incidences of LEL according to the risk scores were calculated from the scoring algorithm (Table 4). Since the cumulative incidence rose with a great difference when the risk score was 13 or higher, we established a cutoff score of 13 and classified patients into two risk groups. As a result, 209 (79.5%) patients were classified as part of the “low-risk” group, and 54 (20.5%) were classified as part of the “high-risk” group. A significant difference was found in the cumulative incidences between low- and high-risk groups (HR, 4.252; Figure 1d). The AUC value of predicting LEL with this risk group classification was 0.675, 0.669, and 0.662 for LEL at 1, 3 and 5 years after surgery.

## 3. Discussion

This retrospective study showed that laparoscopic surgery, harvesting more than 30 pelvic lymph nodes, and PALND were associated with a higher incidence of LEL, and that adjuvant pelvic EBRT has a negative impact on LEL in propensity matched analysis compared to vaginal brachytherapy in gynecologic cancer patients. In addition, we constructed a LEL risk score model to estimate the probability of LEL after adjuvant RT and demonstrated a significant difference between the high- and low-risk groups determined by the risk score. 

Determining the diagnostic criteria for LEL is an important outstanding issue because there is no gold-standard method and no consensus as to the optimal method for assessing LEL. Nevertheless, circumferential measurement has been the traditional and widely used method in previous studies [10,16,17]. Therefore, we adopted the diagnostic criteria of a ≥2 cm difference in circumferential measurement, which is routinely performed at our lymphedema clinic. 

The reported incidence of LEL varies widely across studies. When focusing on patients who received adjuvant RT, the rate of clinically diagnosed LEL was higher in previous reports than the present study. These rates were 21.1% for endometrial cancer [14] and 37.9% for gynecologic cancer [9]. In this study, most cases of LEL development were found within 3 years after surgery and especially more commonly within the first year, which was similar to the results of previous studies [14,18]. However, previous studies with long-term follow-ups revealed that some patients developed LEL at five years after RT [19] and even 10 years after surgery [10], indicating the importance of long-term follow-up for LEL.

Among the risk factors identified in this study, the number of harvested lymph nodes and PALND were consistent with the results of previous reports. The cutoff value of > 30 for the number of harvested lymph nodes in this study was similar to that of previous studies [9,11,20]. We only counted the pelvic lymph nodes, but less than one third of the patients underwent PALND and the mean number of harvested para-aortic lymph nodes was 2.3 in the full patient cohort. In addition, we demonstrated that the type of surgical approach was associated with LEL development. Whether or not LEL is influenced by the type of surgical approach has been controversial. Barnett et al. [21] reported a higher incidence of LEL in endometrial cancer patients after laparoscopic hysterectomy plus pelvic lymph node dissection (PLND) compared to laparotomy (10.6% vs. 1.6%). Kuoppala et al. [22] also reported similar results (12.5% vs. 7.5%). This may be due to longer surgery time, greater number of lymph nodes removed and increased insult to the lymphatics accompanying laparoscopy. Barnett et al. suggested that with the increased surgical view magnification available using the laparoscopic approach, surgeons may more aggressively dissect to distal lymph nodes, including circumflex iliac nodes distal to the external iliac nodes, the dissection of which has been found to cause LEL [13,20]. However, other studies failed to show an association between surgical approach and LEL [9,14].

Compared to vaginal brachytherapy, adjuvant pelvic EBRT was reported to cause more complications and impair long-term quality of life [15,23,24]. In addition, it is commonly expected that pelvic EBRT would contribute more to postoperative LEL than vaginal brachytherapy. Indeed, the incidence of lymphedema after adjuvant vaginal brachytherapy ranges from 3.7% to 11.1% [23,25], which is relatively low compared to 25.5% to 71.4% after adjuvant pelvic EBRT [4,10], except one study that reported lower incidence of LEL after pelvic EBRT than after vaginal brachytherapy [14]. However, to our knowledge, this is the first study to perform direct comparison of the risk of LEL following pelvic EBRT and vaginal brachytherapy and to show that pelvic EBRT is associated with higher risk of postoperative LEL.

This is also the first study to suggest a prediction model for LEL after adjuvant RT in gynecologic cancer patients. The prediction power of the model in this study was comparable to the AUC value of 0.64 at five years after PLND in gynecologic cancer patients in a previous study [12]. Our study provides a useful model that could be implemented in clinical settings or may be improved with further subsequent studies based on the present study.

Since the present study is retrospective in nature, several limitations exist. First, some potential risk factors of LEL could not be fully investigated, such as medication history including nonsteroidal anti-inflammatory drugs, and the detailed site of lymph node dissection. Second, as preoperative assessments of the limb volume or lymphatics were not performed routinely, bilateral lymphedema was hard to distinguish from systemic edema and accurate diagnosis with circumferential measurements could not be made. Therefore, we did not analyze bilateral lymphedema in this study and it is possible that some patients with bilateral LEL were not identified. Additionally, lymph flow alteration could not be evaluated due to the lack of preoperative assessments and therefore, we expect further study evaluating the lymph flow alteration in high risk patients of this study could enhance the result of this study. Lastly, some bias may be present according to RT modality because patients with earlier stage were treated more often with brachytherapy alone and the number of patients treated with brachytherapy alone was small for a detailed analysis. Nevertheless, we attempted to overcome these limitations using statistical methods such as propensity score matching to draw clinically meaningful results.

## 4. Materials and Methods

### 4.1. Patients

We retrospectively analyzed a total of 263 International Federation of Gynecology and Obstetrics (FIGO) stage I–III gynecologic cancer patients who underwent adjuvant RT after hysterectomy as the initial treatment. All patients in this study underwent pelvic EBRT or vaginal brachytherapy between January 2013 and December 2017 at our institution. Patients with a minimum follow-up of 1-year without cancer recurrence or progression were included. Patients with a history of deep vein thrombosis or acute postoperative lymphedema, which typically begins within 4 to 6 weeks after surgery and before the course of RT, were excluded. Follow-up was censored when there was any evidence of cancer recurrence or progression on radiologic exams or biopsy. 

### 4.2. Treatment

The extent of lymph node dissection was determined by the surgeon’s assessment of potential for lymph node metastasis. Similarly, the adjuvant RT and RT modality was determined by the radiation oncologists’ assessment of the potential and area of risk for locoregional recurrence based on patient and tumor characteristics such as age, stage, tumor size, histologic grade, and lymphovascular space invasion, in addition to preoperative clinical tumor characteristics.

Adjuvant pelvic EBRT was delivered with either 3D-conformal radiation therapy or intensity-modulated radiation therapy. The most frequently prescribed and median dose for EBRT was 50.4 Gy in 28 fractions. In all EBRT cases, the whole pelvis including iliac chains was covered in the RT field, and if involved in pathologic examination, the para-aortic lymph nodes were also included in the field. In brachytherapy, a median dose of 30 Gy in 5 fractions was delivered to the vaginal vault with an iridium-192 source.

Adjuvant chemotherapy was administered before, concurrently, or after RT, or with a combination of schedules. Cisplatin alone was the most frequently used regimen for concurrent chemoradiotherapy. Some of the patients with advanced stage were treated with sequential chemotherapy after concurrent chemoradiotherapy and the most frequently used regimen was paclitaxel plus carboplatin.

### 4.3. Diagnosis of LEL

In our institution, gynecologic oncologists, radiation oncologists, and lymphedema specialists cooperate to accurately diagnose and appropriately manage LEL. During follow-up, patients were questioned or examined by gynecologic oncologists or radiation oncologists to identify lower extremity edema. If lower extremity edema was present, patients were referred to lymphedema specialists, and circumferential measurement was performed. Circumferences were measured at five points or more, including the knee joint line as well as 10 cm and 20 cm above and below the knee. Patients were diagnosed with LEL if the circumference difference was 2 cm or greater at any of the measured points and other causes of lower extremity edema such as vascular or systemic edema were ruled out. If the diagnosis of LEL was uncertain with only circumferential measurements, other tests including perometry, lymphoscintigraphy, lymphangiography, and ultrasound sonography were conducted to confirm the diagnosis. Ultrasound sonography was useful for measuring the volume of each extremity and ruling out deep vein thrombosis when suspected. However, those were not performed routinely and were only used auxiliary. Therefore, we defined circumferential difference ≥ 2 cm as LEL in this study and analyses were conducted based on this definition. Patients with central, suprapubic, or inguinal lymphedema were not included for analysis. 

### 4.4. Statistical Analysis

Patient characteristics were compared between the patients treated with EBRT regardless of subsequent brachytherapy (EBRT group) and those with brachytherapy alone (brachytherapy group) using Chi-square test and Fisher’s exact test, as appropriate. In order to control for differences in characteristics between the two groups, we used the propensity score matching method. Propensity scores were generated using variables available in our medical records from among the risk factors identified in previous studies [12,14,17,19] and in this study. The selected variables were age, preoperative body mass index, lymph node metastasis, adjuvant chemotherapy, PALND, surgical approach, and number of harvested pelvic lymph nodes. Using propensity scores, the EBRT group and brachytherapy group were matched with a 1:1 nearest-neighbor matching protocol with a caliper width of 0.2 standard deviations.

Actuarial cumulative incidences of LEL were calculated using the Kaplan-Meier method and were compared by log-rank test. Univariate and multivariate analyses were executed with Cox proportional hazards model to identify the risk factors associated with LEL. All variables with *p* values < 0.1 in the univariate analysis were included in the multivariate analysis. All statistical analyses were conducted in R version 3.5.0, and propensity score matching was conducted with the MatchIt package (version 3.0.2).

### 4.5. Ethics Statement

This study was approved by the Institutional Review Board of our institution (H-1804-093-938). Informed consents were not obtained from the patients as this was a retrospective study.

## 5. Conclusions

We found that harvesting more than 30 pelvic lymph nodes, para-aortic lymph node dissection, laparoscopic surgery, and pelvic EBRT were associated with a higher incidence of LEL in gynecologic cancer patients treated with adjuvant RT. Combining these risk factors, we developed a prediction model to estimate the probability of LEL. The high-risk group identified with this model may require closer observation for LEL in order to facilitate early diagnosis and management.

## Figures and Tables

**Table 1:** Patient characteristics in the entire cohort and matched cohort.

Variable	Entire Cohort (Before Matching)		*p*	Matched Cohort (After Matching)		*p*
	Brachytherapy	EBRT ± Brachytherapy		Brachytherapy	EBRT ± Brachytherapy	
	(*n* = 42)	(*n* = 221)		(*n* = 37)	(*n* = 37)	
Age (years)			0.007 *			0.804*
< 55	11 (26.2%)	111 (50.2%)		11 (29.7%)	13 (35.1%)	
≥ 55	31 (73.8%)	110 (49.8%)		26 (70.3%)	24 (64.9%)	
Preoperative BMI (kg/m^2^)			0.698 †			1.000 *
< 25	24 (57.1%)	131 (59.3%)		24 (64.9%)	23 (62.2%)	
≥ 25	18 (42.9%)	84 (38.0%)		13 (35.1%)	14 (37.8%)	
Unknown	0 (0.0%)	6 (2.7%)		0 (0.0%)	0 (0.0%)	
Hypertension	19 (45.2%)	54 (24.4%)	0.010 *	15 (40.5%)	7 (18.9%)	0.075 *
Diabetes mellitus	8 (19.0%)	22 (10.0%)	0.151 *	6 (16.2%)	4 (10.8%)	0.736 †
Chronic kidney disease	3 (7.1%)	3 (1.4%)	0.054 †	3 (8.1%)	0 (0.0%)	0.240 †
Peripheral vascular disease	1 (2.4%)	3 (1.4%)	0.504 †	1 (2.7%)	2 (5.4%)	1.000 †
Lower extremity surgery history	5 (11.9%)	15 (6.8%)	0.407 *	4 (10.8%)	2 (5.4%)	0.674 †
Origin of cancer			<0.001 †			0.007 †
Cervix	5 (11.9%)	121 (54.8%)		4 (10.8%)	15 (40.5%)	
Endometrium	37 (88.1%)	88 (39.8%)		33 (89.2%)	22 (59.5%)	
Uterine sarcoma	0 (0.0%)	7 (3.2%)		0 (0.0%)	0 (0.0%)	
Double/Triple primary	0 (0.0%)	5 (2.3%)		0 (0.0%)	0 (0.0%)	
FIGO stage (2009)			0.002 †			1.000†
I–II	42 (100.0%)	178 (80.5%)		37 (100.0%)	36 (97.3%)	
III	0 (0.0%)	39 (17.7%)		0 (0.0%)	0 (0.0%)	
Unknown	0 (0.0%)	4 (1.8%)		0 (0.0%)	1 (2.7%)	
Lymph node metastasis	0 (0.0%)	74 (33.5%)	<0.001 †	37 (100.0%)	34 (91.9%)	0.240 †
Surgical approach			0.001 *			0.754 †
Open	5 (11.9%)	87 (39.4%)		5 (13.5%)	5 (13.5%)	
Laparoscopy	37 (88.1%)	133 (60.2%)		32 (86.5%)	31 (83.8%)	
Vaginal	0 (0.0%)	1 (0.5%)		0 (0.0%)	1 (2.7%)	
Number of pelvic LNs harvested			0.376 *			1.000 *
≤ 30	36 (85.7%)	173 (78.3%)		31 (83.8%)	32 (86.5%)	
> 30	6 (14.3%)	48 (21.7%)		6 (16.2%)	5 (13.5%)	
PALND	14 (33.3%)	71 (32.1%)	1.000 *	14 (37.8%)	15 (40.5%)	1.000 *
Adjuvant chemotherapy	2 (4.8%)	151 (68.3%)	<0.001 †	2 (5.4%)	2 (5.4%)	1.000 †

EBRT: External beam radiation therapy; BMI: Body mass index; FIGO: International Federation of Gynecology and Obstetrics; LN: Lymph node; PALND: Para-aortic lymph node dissection; * Chi-square test; † Fisher’s exact test.

**Table 2:** Multivariate analysis of risk factors associated with lower extremity lymphedema.

Variable	Univariate		Multivariate	
	HR (95% CI)	*p* *	HR (95% CI)	*p* *
Age ≥ 55 years	1.207 (0.617–2.358)	0.581		
Preoperative BMI ≥ 25 kg/m^2^	0.636 (0.306–1.325)	0.227		
Hypertension	0.774 (0.352–1.705)	0.525		
Diabetes mellitus	0.223 (0.301–1.630)	0.139		
History of lower extremity surgery	0.346 (0.047–2.529)	0.346		
Endometrial cancer	1.590 (0.814–3.109)	0.175		
Lymph node metastasis	1.167 (0.572–2.383)	0.672		
Advanced FIGO stage	1.217 (0.505–2.933)	0.661		
Laparoscopic surgery	2.034 (0.923–4.482)	0.078	2.548 (1.131–5.740)	0.024
Number of pelvic LNs harvested > 30	2.106 (1.048–4.233)	0.037	2.246 (1.093–4.616)	0.028
PALND	2.391 (1.232–4.641)	0.010	2.305 (1.180–4.502)	0.014
EBRT ± Brachytherapy	1.969 (0.602–6.439)	0.262		
Adjuvant chemotherapy	0.744 (0.383–1.444)	0.382		

HR: Hazard ratio; CI: Confidence interval; BMI: Body mass index; FIGO: International Federation of Gynecology and Obstetrics; LN: Lymph node; PALND: Para-aortic lymph node dissection; EBRT: External beam radiation therapy; * Cox proportional hazards model.

**Table 3:** Coefficient of risk factors and lower extremity lymphedema risk scoring.

Variable	HR *	Coefficient *	Risk Score
Laparoscopic surgery	2.738	1.007	+5
Number of pelvic LNs harvested > 30	2.137	0.759	+4
PALND	2.291	0.829	+4
EBRT ± Brachytherapy	2.258	0.814	+4

LN: Lymph node; PALND: Para-aortic lymph node dissection; EBRT: External beam radiation therapy. * Cox proportional hazards model.

**Table 4:** Cumulative incidences of lower extremity lymphedema according to risk scores.

Risk Score	*n*	%	1-Year (%)	3-Year (%)	5-Year (%)
0	2	0.8	0.0	0.0	0.0
4	48	18.3	2.1	5.1	5.1
5	21	8.0	0.0	8.3	8.3
8	29	11.0	10.3	14.8	14.8
9	95	36.1	8.4	8.4	13.0
12	14	5.3	14.3	14.3	14.3
13	45	17.1	26.7	32.5	32.5
17	9	3.4	33.3	33.3	33.3

## References

[1] Rowlands I.J., Beesley V.L., Janda M., Hayes S.C., Obermair A., Quinn M.A., Brand A., Leung Y., McQuire L., Webb P.M. (2014). Quality of life of women with lower limb swelling or lymphedema 3–5 years following endometrial cancer. Gynecol. Oncol..

[2] Greimel E.R., Vlasic K.K., Waldenstrom A.C., Duric V.M., Jensen P.T., Singer S., Chie W., Nordin A., Radisic V.B., Wydra D. (2006). The European Organization for Research and Treatment of Cancer (EORTC) Quality-of-Life questionnaire cervical cancer module: EORTC QLQ-CX24. Cancer.

[3] Mendivil A.A., Rettenmaier M.A., Abaid L.N., Brown J.V., Micha J.P., Lopez K.L., Goldstein B.H. (2016). Lower-extremity lymphedema following management for endometrial and cervical cancer. Surg. Oncol..

[4] Yost K.J., Cheville A.L., Al-Hilli M.M., Mariani A., Barrette B.A., McGree M.E., Weaver A.L., Dowdy S.C. (2014). Lymphedema after surgery for endometrial cancer: Prevalence, risk factors, and quality of life. Obstet. Gynecol..

[5] Casley-Smith J.R. (1995). Alterations of Untreated Lymphedema and It Grade Over Time. Lymphology.

[6] Eby C.S., Brennan M.J., Fine G. (1967). Lymphangiosarcoma: A Lethal Complication of Chronic Lymphedema: Report of Two Cases and Review of the Literature. Arch. Surg..

[7] Huey G.R., Stehman F.B., Roth L.M., Ehrlich C.E. (1985). Lymphangiosarcoma of the edematous thigh after radiation therapy for carcinoma of the vulva. Gynecol. Oncol..

[8] Park H.J., Kim H.J., Wu H.-G., Kim H., Ha S.W., Kang S.-B., Song Y.-S., Park N.-H., Kim J.-W. (2011). The influence of adjuvant radiotherapy on patterns of failure and survivals in uterine carcinosarcoma. Radiat. Oncol. J..

[9] Kim M., Suh D.H., Yang E.J., Lim M.C., Choi J.Y., Kim K., No J.H., Kim Y.-B. (2017). Identifying risk factors for occult lower extremity lymphedema using computed tomography in patients undergoing lymphadenectomy for gynecologic cancers. Gynecol. Oncol..

[10] Kim J.H., Choi J.H., Ki E.Y., Lee S.J., Yoon J.H., Lee K.H., Park T.C., Park J.S., Bae S.N., Hur S.Y. (2012). Incidence and risk factors of lower-extremity lymphedema after radical surgery with or without adjuvant radiotherapy in patients with FIGO stage I to stage IIA cervical cancer. Int. J. Gynecol. Cancer.

[11] Bae H.S., Lim M.C., Lee J.S., Lee Y., Nam B.H., Seo S.-S., Kang S., Chung S.H., Kim J.-Y., Park S.-Y. (2016). Postoperative Lower Extremity Edema in Patients with Primary Endometrial Cancer. Ann. Surg. Oncol..

[12] Kuroda K., Yamamoto Y., Yanagisawa M., Kawata A., Akiba N., Suzuki K., Naritaka K. (2017). Risk factors and a prediction model for lower limb lymphedema following lymphadenectomy in gynecologic cancer: A hospital-based retrospective cohort study. BMC Womens Health.

[13] Ohba Y., Todo Y., Kobayashi N., Kaneuchi M., Watari H., Takeda M., Sudo S., Kudo M., Kato H., Sakuragi N. (2011). Risk factors for lower-limb lymphedema after surgery for cervical cancer. Int. J. Clin. Oncol..

[14] Beesley V.L., Rowlands I.J., Hayes S.C., Janda M., O’Rourke P., Marquart L., Quinn M.A., Spurdle A.B., Obermair A., Brand A. (2015). Incidence, risk factors and estimates of a woman’s risk of developing secondary lower limb lymphedema and lymphedema-specific supportive care needs in women treated for endometrial cancer. Gynecol. Oncol..

[15] Karabuga H., Gultekin M., Tulunay G., Yuce K., Ayhan A., Yuce D., Yildiz F. (2015). Assessing the quality of life in patients with endometrial cancer treated with adjuvant radiotherapy. Int. J. Gynecol. Cancer.

[16] Hopp E.E., Osborne J.L., Schneider D.K., Bojar C.J., Uyar D.S. (2016). A prospective pilot study on the incidence of post-operative lymphedema in women with endometrial cancer. Gynecol. Oncol. Rep..

[17] Deura I., Shimada M., Hirashita K., Sugimura M., Sato S., Sato S., Oishi T., Itamochi H., Harada T., Kigawa J. (2015). Incidence and risk factors for lower limb lymphedema after gynecologic cancer surgery with initiation of periodic complex decongestive physiotherapy. Int. J. Clin. Oncol..

[18] Ryan M., Stainton M.C., Slaytor E.K., Jaconelli C., Watts S., Mackenzie P. (2003). Aetiology and prevalence of lower limb lymphoedema following treatment for gynaecological cancer. Aust. New Zeal. J. Obstet. Gynaecol..

[19] Mitra D., Catalano P.J., Cimbak N., Damato A.L., Muto M.G., Viswanathan A.N. (2016). The risk of lymphedema after postoperative radiation therapy in endometrial cancer. J. Gynecol. Oncol..

[20] Todo Y., Yamazaki H., Takeshita S., Ohba Y., Sudo S., Minobe S., Okamoto K., Kato H. (2015). Close relationship between removal of circumflex iliac nodes to distal external iliac nodes and postoperative lower-extremity lymphedema in uterine corpus malignant tumors. Gynecol. Oncol..

[21] Barnett J.C., Havrilesky L.J., Bondurant A.E., Fleming N.D., Lee P.S., Secord A.A., Berchuck A., Valea F.A. (2011). Adverse events associated with laparoscopy vs. laparotomy in the treatment of endometrial cancer. Am. J. Obstet. Gynecol..

[22] Kuoppala T., Tomás E., Heinonen P.K. (2004). Clinical outcome and complications of laparoscopic surgery compared with traditional surgery in women with endometrial cancer. Arch. Gynecol. Obstet..

[23] Atlan D., Touboul E., Deniaud-Alexandre E., Lefranc J.-P., Antoine J.-M., Jannet D., Lhuillier P., Uzan M., Huart J., Genestie C. (2002). Operable Stages IB and II cervical carcinomas: A retrospective study comparing preoperative uterovaginal brachytherapy and postoperative radiotherapy. Int. J. Radiat. Oncol. Biol. Phys..

[24] Nout R.A., Putter H., Jürgenliemk-Schulz I.M., Jobsen J.J., Lutgens L.C.H.W., Van Der Steen-Banasik E.M., Mens J.W.M., Slot A., Stenfert Kroese M.C., Nijman H.W. (2012). Five-year quality of life of endometrial cancer patients treated in the randomised Post Operative Radiation Therapy in Endometrial Cancer (PORTEC-2) trial and comparison with norm data. Eur. J. Cancer.

[25] Kumar V.J., Nin C.Y., Kuei L.Y., Tan K.H.S., Yeo R., Lam P.Y.K. (2010). Survival and disease relapse in surgical stage I endometrioid adenocarcinoma of the uterus after adjuvant vaginal vault brachytherapy. Int. J. Gynecol. Cancer.

